# An Innovative Methodology to Characterize, at the Molecular Scale, Interactions in Polysaccharide Aqueous Solutions

**DOI:** 10.3390/molecules29081787

**Published:** 2024-04-15

**Authors:** Alexandre Cordinier, Igor Petukhov, Nicolas Hucher, Michel Grisel

**Affiliations:** Université Le Havre Normandie, Normandie Univ, URCOM UR 3221, F-76600 Le Havre, France; alexandrecordinier@ymail.com (A.C.);

**Keywords:** interactions, polymers, polysaccharides, H-bonds, hydrophobic mico-domains, dipole-dipole interactions

## Abstract

Characterizing molecular interactions at the microscopic level remains difficult and, therefore, represents a key target to better understand macromolecule and biomacromolecule behaviors in solution, alone, or in mixtures with others. Therefore, accurate characterization in liquid media, especially in aqueous solutions, without causing any perturbation of the system in which they are studied, is quite difficult. To this purpose, the present paper describes an innovative methodology based on fluorescence spectrophotometry. Two molecular fluorescent probes, namely 8-anilino-1-naphtalenesulfonic acid (ANS) and 2-benzofuryl-3-hydroxy-4(1H)-quinolone (3HQ-Bf), were selected to characterize, respectively, the dipole-dipole interactions and hydrophobic micro-domains, for the first one, and hydrogen bonding, for the second. As a support to study molecular interactions, xanthan, galactomannan, and corresponding mixtures of these substances which are well known to exhibit a synergy of interactions in well-defined mixture conditions were chosen. Once the methodology was set up, the existence of the three types of interactions in these systems was demonstrated, thus allowing the elucidation of the mechanisms of interactions at the molecular scale.

## 1. Introduction

Molecular interactions are essential to maintain cohesion between different compounds in materials or biological phenomena such as enzyme/substrate complex formations [1]. In solution, molecular interactions are non-covalent and are essentially dependent on the distance of the interaction between the chemical entities or groups. They can be either intramolecular, like for α-helix in secondary structure proteins [2], or intermolecular interactions, such as between two DNA strands [3]. Among the different types of non-covalent interactions, H-bonds, dipole-dipole (also named van der Waals) interactions, in which are included the creation of hydrophobic micro-domains, and electrostatic interactions are by far the most frequent molecular phenomena which are encountered in aqueous polymer media [4,5,6]. A precision definition should be made about the term hydrophobic micro-domains, which are often referenced as hydrophobic interactions in the literature. The creation of such hydrophobic domains is mainly due to a reorganization of water molecules [7,8] solvating apolar hydrophobic parts of a solute in aqueous solution.

Nowadays, there is no reliable methodology allowing scientists to fully characterize the molecular interactions within polymer aqueous solutions without strongly disturbing the system. As an example, chaotropic agents, such as urea [5] or guanidine hydrochloride [9], are often used to show the presence of H-bonds; unfortunately, these compounds strongly interfere with the H-bond network by “breaking” the H-bonds, hence the name of “H-bond breakers”, which is also used for this class of chemical agents. Another way to show the presence of H-bonds is to analyze the characteristic vibration bands in FTIR spectra [10]; to this end, sample preparation is necessary by removing the liquid from the sample, often using freeze drying, thus making the system highly different from the solution’s initial state. Another striking example is dipole-dipole interactions, which can be monitored by atomic force microscopy [11] (AFM); however, this powerful technique is not directly applicable in the liquid state. Highlighting electrostatic interactions is generally conducted by studying the effect of the absence or the presence of salts in polyelectrolyte solutions via, for example, potentiometric titrations [12,13]. In the field of nanolithography, AFM allows the observation and the direct localization of hydrophobic domains due to the self-assembly phenomena of amphiphilic block copolymers [14]. Such hydrophobic domains have also been observed through cryo-TEM images for self-assembled amphiphilic objects, such as surfactants or amphiphilic bloc copolymers dispersed in solution [15,16]. Then again, sample preparation, such as freeze drying, is required for these images, which may cause disruptions to interactions, thus making it necessary to develop non-disturbing tools for accurately investigating molecular interactions.

Among other techniques, fluorescence spectroscopy is a promising technique based on the use of small organic fluorescent dyes. It has already been widely used to study molecular interactions in phospholipid bilayers [17] to distinguish dipole-dipole interactions and hydrogen bonds, in organic solvent mixtures [18] to detect and quantify alcohol components, and in aqueous protein media [19,20] to determine surface proteins’ hydrophobicity. Thus, fluorescent probes are advantageously sensitive to one or several specific interactions [21] and can be interestingly present in free diffusion in a system.

**Figure 1:**
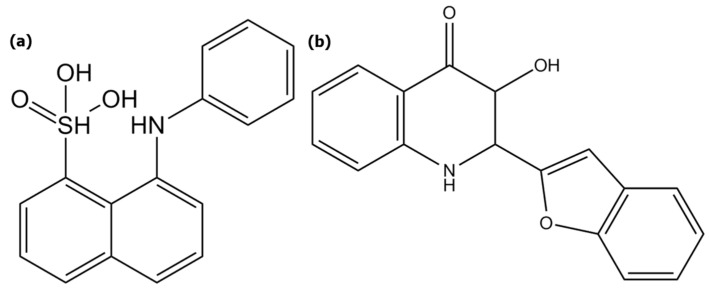
Chemical structures of (**a**) 8-anilino-1-naphtalensulfonic acid (ANS) and (**b**) 2-benzofuryl-3-hydroxy-4(1H)-quinolone (3HQ-Bf).

As an example, 8-anilino-1-naphtalensulfonic acid (ANS) (Figure 1a) is a fluorescent probe mainly used in its basic form which is specific to polar interactions and fits directly in hydrophobic micro-cavities (or micro-domains). There are several fluorescent probes with similar fluorescent properties, such as 6-propionyl-2-dimethylaminonaphthalen [22], 1-N-Phénylnapththylamine [23], and 2-anilinonaphthalen-6-sulfonic [24] acid, also known, respectively, as PRODAN, NPN, and 2,6-ANS, on which our tests were realized. At the end, ANS was selected because of its fluorescent properties, which are the least disturbed by the analytical conditions. Its fluorescent quantum yield is ten- to a hundred-fold higher when ANS is surrounded by a protein compared to pure water [20,25] or on protein surfaces [19]. It has been used to identify specific sites in proteins such as bovine serum albumin [20] (BSA), apomyoglobin, and apohemoglobine [26]. ANS has also been applied for a long time in order to study biological membranes [25] and membrane type structures [27,28,29]. Stryer and co-workers [26] demonstrated that the fluorescent properties of ANS are strongly dependent on medium polarity [30,31]. There is, indeed, a blue shift of the wavelength at the maximum emission intensity (EI) as long as the solvent is non-polar. Therefore, ANS can advantageously communicate information related to dipole-dipole interactions. Finally, Haynes and Staerk [32] showed that ANS fluorescence quantum yield is not dependent on the pH within a range from from 3 to 10 and is not disturbed by the presence of salts in solution. As it has been described above, the ANS probe has been used in many studies whose subjects were related to proteins but not in studies dealing with natural water-soluble polymers.

In addition, 2-benzofuryl-3-hydroxy-4(1H)-quinolone, also denoted as 3HQ-Bf, functions as a probe (Figure 1b) that is sensitive to hydrogen bonding. It was developed by Bilokin’ and co-workers [33]; it has an excited state due to intramolecular proton transfer (ESIPT) [33,34] that induces both dual fluorescence and ratiometric probe properties. In particular, the ratio between the EI of the N* (1N* and 2N*) bands and the EI of the T* (EI_N*_/EI_T*_) bands is affected by the relative density of the hydrogen bond network; the more intense the hydrogen bond network, the higher the value of the ratio EI_N*_/EI_T*_. In his work, Bilokin’ clearly established that EI_N*_/EI_T*_ is correlated to Abraham’s parameter β, a parameter initially introduced by Kamlet and Taft [35]. Based on this β parameter, they developed a hydrogen bond basicity (hydrogen bond acceptor ability, HBA) scale which was further developed and completed by Abraham’s works [36,37]. Simply speaking, the higher the EI_N*_/EI_T*_ value is, the richer the environment of hydrogen bonds, this latter corresponding to a higher hydrogen bond density.

In this paper, an original methodology is presented for characterizing hydrophobic micro-domains, dipole-dipole interactions, and the H-bond network which can be developed in polysaccharide solutions and mixtures in aqueous medium. To achieve this goal, a model system consisting of two polysaccharides, namely xanthan gum (X) and galactomannans (GM), was prepared and used as a support to study the molecular interactions particularly when both gums are mixed in specific conditions.

## 2. Experimental Section

### 2.1. Materials

The fluorescence probe 2-benzofuryl-3-hydroxy-4(1H)-quinolone (3HQ-Bf) was prepared following a previously described procedure [33]. The 8-anilino-1-naphtalensulfonic acid (ANS) probe and the bactericide sodium azide (NaN_3_) were provided by Sigma-Aldrich (Saint Quentin Fallavier, France). Na_2_HPO_4_ was provided by Rhône-Poulenc (lyon, France) and NaCl was provided by Labogros (Coueron, France). Methanol for HPLC was provided by Carlo Erba (Val de Reuil, France) and ultra-pure water (18.2 mΩ resistivity) was obtained from an Easypure UV Compact ultrapure water system (Thermo Scientific Barnstead, Illkirch, France). Both polysaccharide powders, locust bean gum (LBG) and xanthan gum (X), were kindly provided by Degussa (Puteaux, France). Guar gum (GG) was provided by Danisco (Neuilly-sur-Seine, France). The main characteristics of the gum samples used in the present work were determined and are listed in Table 1.

### 2.2. Preparation of Probe Stock Solution

ANS powder was dissolved in ultra-pure water under magnetic stirring for at least 6 h until total solubilization. 3HQ-Bf powder was first pre-dispersed in methanol above its solubility limit under magnetic stirring for at least 6h. Then, the mixture was centrifuged at 500× *g* for 15 min and only the supernatant solution was kept. The probe stock solution concentrations were checked by UV–visible analysis (UV-1800, Shimadzu, Marne-la-Vallée, France); the UV optic density band at 350 nm (ANS) had a molar extinction coefficient ε equal to 4.95 × 10^3^ M^−1^·cm^−1^ [42] and the UV optic density band at 384 nm (3HQ-Bf) had a molar extinction coefficient ε equal to 17.7 × 10^3^ M^−1^·cm^−1^ [33].

### 2.3. Preparation of Polysaccharide Solutions

The xanthan gum powder (X) was purified beforehand by the supplier and was used as received. X powder was gradually dissolved under mechanical stirring for at least 6 h in a phosphate buffer (pH 7.4; Na_2_HPO_4_ at 0.02 M) to fix the pH. NaCl 0.01 M was added to keep the xanthan in its native form. The bactericide NaN_3_ (400 ppm) was added to prevent bacterial degradation. X stock solution dry–solid content was determined with a thermal gravimetric analysis (TGA; Setsys TGA 1200, SETARAM, Caluire, France) by dehydrating a sample of the X stock solution (40 to 60 mg) and collecting at the end of the analysis the specific masses of the dehydrated sample and the ashes. The X stock solution was then kept for a maximum of 2 months at 4 °C. Each galactomannan gum (LBG, GG) powder was dispersed at 83 °C for 3 h in ultra-pure water under mechanical stirring. Then, an additional 3 h of mechanical stirring at room temperature in a water bath was performed and 400 ppm of NaN_3_ was added. Then, the LBG and GG stock solutions were centrifuged at 14,000× *g* for 1 h as many times as necessary to remove non-soluble residues. The extracts were dried using an oven (102 °C), which allowed the determination of the final galactomannan stock solutions’ concentration of supernatant. Galactomannan stock solutions were then kept for a maximum of 2 months at 4 °C. A total of 40 g of X/GM mixtures were prepared from diluted solutions from each polysaccharide stock solution to get the desired total polysaccharide final concentrations. X/GM mixtures were magnetically stirred for at least 20 min in a glass beaker and then centrifuged at 5000× *g* for 5 min at 20 °C to remove air bubbles.

### 2.4. Fluorescence Measurements

Fluorescence experiments were carried out at a steady state on a CARY VARIAN fluorescence spectrophotometer using a temperature controller Peltier device (Agilent Technologies, Massy, France). Once centrifuged, each polysaccharide solution and X/GM mixture was split into two 15 g fractions: a control fraction (polysaccharides in phosphate buffer) and a sample fraction; this latter was added to a precise amount of florescent probe (ANS or 3HQ-bf) stock solution.

Both samples were then homogenized through high strength shear using a micropipette (VWR; adjusted at 4 mL), applying 15 successive sucks/releases. Then, approximately 3 mL of each fraction were put into two fluorescence quartz cells (Hellma). Fluorescence spectra were registered with and without probes to allow for the correction of background noise. In addition, in order to get usable and statistically reliable probe signals, five successive scans were performed per measurement; each measurement was performed in triplicate to minimize random fluorescence signals due to instrumental background noise.

Photo-stability tests were carried out for both ANS and 3HQ-Bf probes with parameters respectively set as followed: T = 20 °C; excitation wavelengths = 390 nm (ANS) and 320 nm (3HQ-Bf); emission scans from 440 nm to 740 nm and from 350 to 700 nm; excitation slit = 5 nm; emission slit = 5 nm; scan speed = 600 nm/min (ANS) and 120 nm/min (3HQ-Bf); photomultiplier = 800 volts. ANS and 3HQ-Bf signals were smoothed using the Savitzky–Golay algorithm.

#### 2.4.1. Hydrophobic Micro-Domains and Dipole-dipole Interactions Analyses Using the ANS Probe

The acquisition parameters were set as follows: T = 20 °C; excitation wavelength = 390 nm; emission scan from 440 nm to 740 nm; excitation slit = 5 nm; emission slit = 5 nm; scan speed = 600 nm/min; photomultiplier = 750 volts. For each sample, five successive scans were acquired to get a mean scan and its standard deviations. Scans were performed just after the homogenization phase and then every 5 min for 1 h, and finally, every hour until signal stabilization. All spectra were corrected and normalized at 740 nm. Data were then fitted with a polynomial fit curve using OriginPro8 SR4 (v8.0951; B951) to get the maxima values (emission intensities (EI) and wavelengths at EI_max_). Statistical analysis was made each time with an ANOVA using Xlstat^®^ (version 14.0.6023.1000, Addinsoft, Paris, France). ANS spectra in different organic solvents were acquired as follows in order to check their dipole-dipole interaction properties: excitation wavelength = 373 nm; emission scan from 440 nm to 740 nm; excitation slit = 1.5 nm; emission slit = 20 nm; scan speed = 120 nm/min; photomultiplier = 720 volts.

#### 2.4.2. H-Bond Network Analyses Using the 3HQ-Bf Probe

The acquisition parameters were fixed as follows: T = 20 °C; excitation wavelength = 320 nm; emission wavelengths = 393 nm (band 1N*) and 414 nm (2N*) and 505 nm, 507 nm, and 511 nm (Band T*) respectively for X/GG 50/50 mixture (0.2% wt), for X/LBG 20/80-30/70-40/60-60/40 X/LBG, and for X/LBG mixtures 0/100-50/50-70/30-80/20-100/0; excitation slit = 5 nm; emission slit = 5 nm; photomultiplier = 800 volts. Each sample was analyzed with five successive analyses to get a mean of the raw IE and its standard deviations at each emission wavelength. Fluorescence analyses were performed just after the homogenization phase, then every 5 min for 1 h, and then every hour until signals stabilized. Wavelength shift checking analyses of λ_1N*_, λ_2N*,_ and λ_T*_ were performed with the same instrumental parameters as described before, and only one scan was registered at t = 0 and t > 24 h. All spectra were corrected and normalized at 700 nm. Statistical analysis was made with an ANOVA using Xlstat^®^ (2016).

### 2.5. Rheological Measurements

Oscillatory shear studies were carried out using a stress-controlled rheometer DHR2 (TA Instruments, Guyancourt, France) equipped with a cone/plate aluminum geometry (60 mm; angle, 2°00′43″; 59 μm) and a Peltier temperature control device. The polysaccharide solutions and mixtures were kept at room temperature for 48 h, and then were analyzed for 3 h. Water loss was prevented by covering the sample with low viscosity silicon oil. The storage (G′) and loss (G″) moduli were measured within the linear viscosity domain at 20 °C. The acquisition parameters were set as follows: T = 20 °C, sampling interval = 10 s/pt, angular frequency (ω) = 1 rad·s^−1^. The same parameters were applied to the analyses checking the viscoelastic properties of the X/GM mixtures with and without fluorescent probes.

## 3. Development of the Methodology for the Determination of Interactions

### 3.1. Probe Responses and Stability Testing

**Figure 2:**
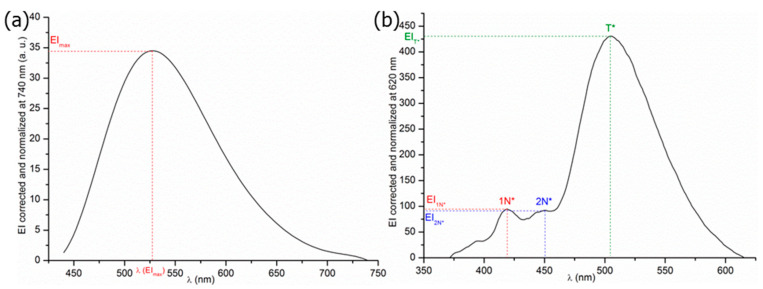
Photobleaching resistance tests of the (**a**) ANS probe and (**b**) 3HQ-Bf probe in phosphate buffer (pH 7.4).

Samples were scanned in a range from 340 to 740 nm at 600 nm·min^−1^ for ANS, while they were scanned in a range from 350 to 700 nm at 120 nm·min^−1^ in the case of 3HQ-Bf. As visible in Figure 2a, the fluorescence properties of ANS remain constant up to 200 scans, thus indicating no probe degradation.

On the contrary, as visible in Figure 2b, a sharp decrease in the 3HQ-Bf fluorescence spectra was observed after 180 scans, whereas there was no change of medium. This is evidence of whitening effects due to an excess of irradiation time exposure. On the basis of this result, it is necessary to limit the samples’ period of exposure for 3HQ-Bf to prevent probe degradation. Thus, only emission intensities (EI) at the specific spectral band wavelengths 1N*, 2N*, and T* were recorded to limit 3HQ-Bf probe photo-instability. We validated the standard deviations of the raw EI of 1N*, 2N*, and T* each time (Table 2).

3HQ-Bf was therefore irradiated five times at the specific band wavelengths of 1N*, 2N*, and T* corresponding to 393, 414, and 507 ± 4 nm (adjusted according to the studied medium), respectively, thus ensuring 3HQ-Bf probe stability over the whole period of the sample measurements. Therefore, both probes’ fluorescent signals were reliable and could be used in the experimental conditions for studying dipole-dipole interactions, hydrophobic micro-domains, and hydrogen bonding in xanthan/galactomannans (X/GMs) mixtures.

### 3.2. Selection and Exploitation of the Probes’ Signal and Intensity

**Figure 3:**
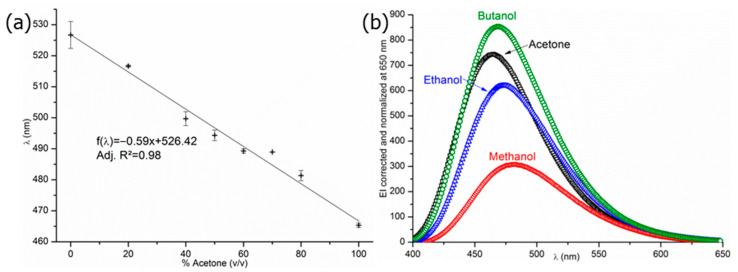
ANS spectrum (**a**) in water/acetone mixtures as a function of %Acetone (*v*/*v*) and (**b**) in different organic solvents.

As described earlier in this paper, the ANS signal is useful as it gives access to two distinct pieces of key information: EI_max_ and wavelength at the maximum emission intensity λ(EI_max_) values. The EI_max_ value of ANS in saline phosphate buffer is equal to 33.7 ± 5.9 arbitrary units (a.u.), this value depending on the instrumental parameters. Its associated wavelength at EI_max_ is equal to 528 ± 2 nm, which is close to the EI_max_ wavelength value in water as determined at 515 nm by Stryer and his team [26]. ANS’ sensitivity to dipole-dipole interactions was examined (Figure 3a) by studying the ANS probe’s behavior in a series of water/acetone mixtures by varying their proportions (*v*/*v*). The results indicate that the EI_max_ wavelength is shifted from 527 nm in pure water to 465 nm in 100% acetone following a linear variation; this is consistent with the “blue shift” previously described by Stryer et al. [26] in water/ethanol mixtures. Additional experiments performed on ANS demonstrated that EI increases with a decrease in the solvent polarity (Figure 3b); again, this is consistent with previous results described in Stryer’s work [19,20,26]. Both results undoubtedly confirm ANS’ sensitivity to medium polarity, and therefore its interest in the present work. Therefore, in the following sections of this paper, variations of the raw EI_ANS_ and a shift of EI_max_ will be respectively interpreted as a variation of the number and the size of the hydrophobic micro-domains, and the presence of dipole-dipole interactions in the studied aqueous medium. Thus interpretations will be made according to the literature previously cited and according to the realized preliminary experiments and tests.

EI_1N*_/EI_T*_ and EI_2N*_/EI_T*_ ratio values are respectively equal to 0.232 ± 0.020 and 0.221 ± 0.010 in saline phosphate buffer. Therefore, both spectral bands 1N* and 2N* appear equivalent. For the following sections of this paper, it was decided to consider only the EI_1N*_/EI_T*_ ratio to investigate the hydrogen bonding occurrence in X/LBG mixtures. In ultra-pure water, an EI_2N*_/EI_T*_ ratio equal to 0.239 ± 0.012 was measured, which is close to the ratio values in phosphate buffer. Nevertheless Bilokin’ [33] determined a value equal to 0.296 in pure water, which appears far from the results obtained herein. Such a significant difference is difficult to explain as information related to the experimental conditions used by these authors is not available. According to Bilokin’s [33] study on 3HQ-Bf and, accordingly, several studies [34,43,44] on dyes with similar chemical structures and also an ESIPT such as 3HQ-Bf, it can be said that 3HQ-Bf is truly sensitive to the H-bond network. Furthermore, thanks to Tomin’s kinetic study [45] on 3-hydroxychromones, a category to which 3HQ-Bf belongs, it has been proved for such dyes that a change of the EI of both spectral bands N* and T* over a period of time is linked to the H-bond network organization, which is linked to the H-bond density in aqueous media. Thus, in the present conditions and being cautious, a change of the value of the EI_1N*_/EI_T*_ ratio through time can be interpreted as the organization or the re-organization of the H-bond network in the studied aqueous medium. The higher the ratio value is, the denser the H-bond network.

### 3.3. Transposition to a Molecular Interaction Study in Polymer Solutions and Mixtures

Depending on the interactions to be characterized, ANS or 3HQ-Bf probes were added to aqueous polymer solutions at molar concentrations of 8 × 10^−6^ M and 5.16 × 10^−6^ M, respectively. The concentrations of both specific probes were set at a low level to avoid modifications at the macroscopic level (change of the macroscopic properties) and also to avoid saturation of the signal, especially in the case of the ANS probe. In the first step, the rheological properties of the polysaccharide solutions and mixtures were measured to check the absence of the probes’ effects on the polymers’ viscoelastic properties, thus ensuring that the probes had no impact (especially plasticizer effect) on the polymers’ structuration and macroscopic properties. Storage moduli in the X/LBG mixtures were, indeed, measured with and without the presence of a molecular probe once both gums were mixed. The storage and loss moduli were identical, thus clearly indicating that the fluorescent molecular probes do not disturb, at this working concentration, the polymer system organization at the macroscopic level. 

#### 3.3.1. Hydrophobic Micro-Domains and Dipole-dipole Interactions

**Figure 4:**
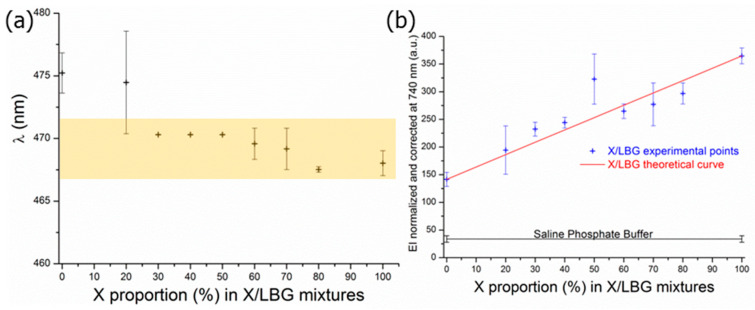
(**a**) Wavelengths at EI_max_ with the background band representing the spectral resolution (5 nm) and (**b**) hydrophobic micro-domain intensity values at equilibrium state as a function of X proportion in X/LBG mixtures at a total polymer concentration of 0.2% (*w*/*w*).

The spectra of xanthan and galactomannan aqueous solutions and mixtures in the presence of ANS were investigated; the wavelengths at the maximum emission intensity (EI_max_) were collected and plotted as a function of the X/LBG ratios. As visible in Figure 4a, there is only a slight decrease in wavelength (7 nm) from X/LBG 0/100 to X/LBG 100/0. The wavelength could be considered as a constant with a mean value of 471 ± 3 nm when considering that the emission split is set at 5 nm. It can therefore be concluded that, in spite of a slight decrease in tendency, there is no significant polarity change of the medium at any X/LBG mixture ratio, thus demonstrating the non-participation of polar interactions between both polysaccharides. On the other hand, one can observe that for each mixture ratio, the EI_ANS_ values are higher than EI_ANS_ as determined in the saline phosphate buffer even for X and LBG pure solutions (Figure 4b); this clearly demonstrates the occurrence of hydrophobic micro-domains related to both polysaccharidic molecular species. 

This original result confirms the occurrence of a self-organization phenomena for each gum in aqueous solution, as was previously suggested by different authors through macroscopic approaches: for xanthan, on the one hand, by Schorsch [46] mainly thanks to polarized microscopy observations; for galactomannan gums, on the other hand, as demonstrated by Rinaudo [47] and Dakia and co-workers [48]; and especially for locust bean gum in dextran/locust bean gum mixtures as studied by Garnier and co-workers [49]. According to Figure 4b, the higher the xanthan proportion in the X/LBG mixture is, the more the hydrophobic micro-domains occur. In addition to that, the EI_ANS_ variation follows a linear evolution, which reflects the absence of synergies, except for the 50/50 X/LBG ratio. Hence, with an EI_ANS_ value significantly deviating from the linear evolution, this proves that X and LBG associate to each other in a specific way around this ratio, which is close to these gums’ optimum synergistic rheological behavior ratio [50,51,52]. Therefore, this shows a clear correlation between molecular hydrophobic micro-domains and macroscopic observations. For the first time, dipole-dipole interactions and hydrophobic micro-domains were characterized at the molecular scale in a complex medium, such as aqueous polymer matrices, through a methodology that can be applied to polysaccharide solutions and mixtures. The experiments suggest that some adjustments may be needed, such as working at a low polymer concentration (0.2% *w*/*w*) or working at a very low concentration of ANS (8 µM).

#### 3.3.2. Hydrogen Bond Network

**Figure 5:**
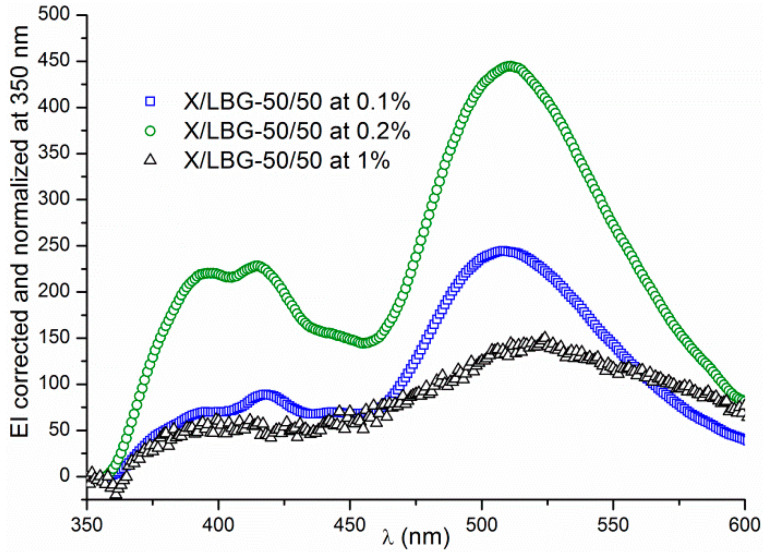
3HQ-Bf in X/LBG mixtures at (**□**) 0.1%; (**○**) 0.2%; and (△) 1% (*w*/*w*).

The hydrogen bond network was investigated with 3HQ-Bf dye. In the first step, a set of experiments was performed for X/LBG 50/50 at different concentrations ranging from 0.1 to 1% *w*/*w* in order to estimate the limit of 3HQ-Bf’s fluorescence properties, especially in terms of polysaccharide concentration. Figure 5 shows that the higher the total polysaccharide concentration, the less intense the EI of the 1N*, 2N*, and T* spectral bands. Furthermore, the signal intensity for the N* bands was too low to be a usable signal.

Thus, band ratios between the 1N* or 2N* bands and T* band cannot be determined properly when the concentration is too high, indicating that the fluorescent properties may be affected by the medium viscosity. Yushchenko and co-workers [44] showed a similar behavior for 2-(2-thienyl)-3-hydroxy-4(1H)-quinolone (3HQT), a fluorescent dye belonging to the same chemical family as 3HQ-Bf. These authors assume that the excitation state T* would be privileged against the excitation state N* thanks to the solvent shell reorganization in a 3HQ-Bf molecule’s environment. This may be responsible for the N* bands’ intensity lowering and then disappearing when the polymer concentration becomes too high.

**Figure 6:**
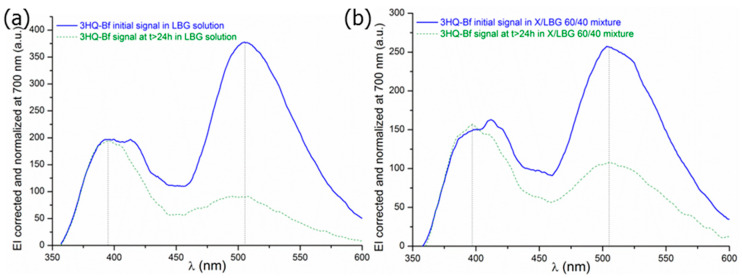
Examples of 3HQ-Bf signals at (**–**) t = 0 and (--) t > 24 h in the (**a**) LBG solution and (**b**) X/LBG 60/40 mixture at 0.2% (*w*/*w*).

As established above, in the present work, the 3HQ-Bf fluorescence signal remains usable for a total polymer concentration up to 0.2% (*w*/*w*) without disturbing the dye ratiometry property. At the beginning, 3HQ-Bf was only a molecule synthesized to specifically evidence hydrogen bonding; thus, using it in aqueous polymeric systems made it necessary to check several parameters. To this end, additional experiments to analyze 3HQ-Bf probe sensitivity to dipole-dipole interactions were envisaged with the aim to check whether the wavelengths (λ_1N*_, λ_2N*_, and λ_T*_) of the three specific bands 1N*, 2N*, and T* are subject to shifts or not. For this purpose, sets of scans were acquired for different X/LBG mixture ratios at t = 0 and at t > 24 h to minimize the irradiation time exposure. In order to keep the results clear, only the results corresponding to the LBG solution and X/LBG 60/40 mixture are reported in Figure 6a and Figure 6b, respectively. As clearly visible on both figures, there is a significant decrease in EI, especially for the T* spectral band between t_0_ and t > 24 h, thus clearly proving that the hydrogen bond network did change. No significant wavelength shifts (vertical black dotted lines) occured over time, meaning that the medium polarity did not change as previously suggested by Bilokin’ and co-workers [33]. These results undoubtedly prove that data acquisition related to 3HQ-Bf probe relies only upon the 1N* band, since the 2N* band is never observed in our case (Figure 6); hence, EI_1N*_ appeared much more reliable and was therefore chosen to calculate the ratio IE_N*_/EI_T*_. Thus, the results exposed in the present paper only consider results based on the 1N* band.

**Figure 7:**
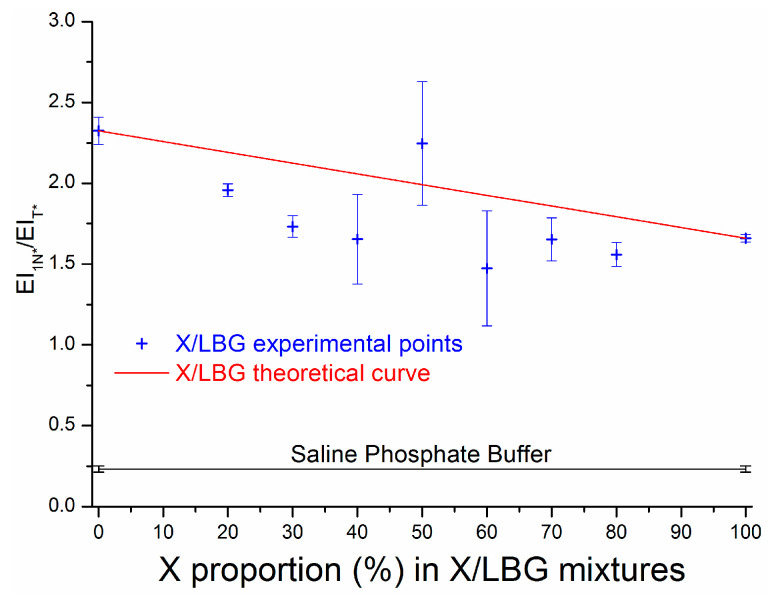
Hydrogen bond network index EI_1N*_/EI_T*_ at equilibrium state as a function of %X in X/LBG mixtures at a total polymer concentration of 0.2% *w*/*w*.

Figure 7 represents the evolution of EI_1N*_/EI_T*_ measured for different X/LBG ratios, including pure X and LBG polysaccharide solutions, for a total polymer concentration of 0.2%. This allows for the observation that whatever the polymer solutions and mixtures, the hydrogen bond network is more developed compared to the solvent alone (saline phosphate buffer). This result is consistent with polysaccharidic molecules that have numerous hydroxyl groups and are thus favorable to the occurrence of hydrogen bonds [47,48,53]. Nevertheless, the hydrogen bond network appears more intense for solutions or mixtures enriched with LBG solution compared to X solution; this result may be explained by different factors: in particular, both xanthan rigidity [54] and polyelectrolyte character are obviously against the establishment of polymer–polymer intra- and inter-molecular interactions. On the other hand, LBG’s flexibility [55] and neutral character may clearly favor molecular interactions through hydrogen bonding.

Undoubtedly hydrogen bonds are favored in LBG pure solution and X/LBG owing to a high LBG/polymer ratio. This result agrees with the previous results determined by Chandrasekaran and co-workers on guaran, a galactomannan gum [56]. On the basis of X-ray scattering experiments combined with numerical simulations, these authors demonstrated that guaran chains interconnect to each other through hydrogen bonds established between the galactose residues of each chain. They assumed that their model could be applied to any kind of galactomannan. Thus, in the case of X/LBG mixtures, it is obvious that X chains disturb the hydrogen bond network, by disrupting the primarily hydrogen bonds which might be located between galactose residues.

Except for the 50/50 X/LBG mixture, it is noteworthy that all the X/LBG mixtures have a EI_1N*_/EI_T*_ ratio significantly lower than the corresponding theoretical values as obtained by calculating the arithmetic mean of the X and LBG combination assuming no synergistic interactions between both gums; this illustrates that the hydrogen bond network organization is not reinforced but more likely to be weakened in the presence of both gums. Thus, X/LBG 50/50 again appears as a remarkable point, both X and LBG gums associate to each other in a very specific conformation strengthening the hydrogen bond network. It is striking that such a specific behavior, corresponding to high interaction intensity, was already observed for hydrophobic micro-domain creation as demonstrated earlier in this paper. Again, and for the first time, the H-bond network was characterized at the molecular scale in a complex medium, such as an aqueous polymer matrix, through a methodology that can also be applied to polysaccharide solutions and mixtures. The experiments also suggest that some adjustments may be required, such as working at a low polymer concentration (0.2% *w*/*w*) or working at very low concentration of 3HQ-Bf (5.16 µM).

#### 3.3.3. Molecular Interactions in an X/LBG Mixture—A Summary

In the presented conditions of analysis and having done multi-parametric tests (photobleaching test, fluorescence properties, etc.), it has been demonstrated on the one hand that a change of EI_ANS_ and a bathochromic shift, respectively, mean a change in the intensity of the hydrophobic micro-domains and the existence of the dipole-dipole interactions. On the other hand, for the 3HQ-Bf probe, any evolution of the EI_1N*_/EI_T*_ ratio value can be interpreted as a change of the organization of the H-bond network, knowing that the higher this ratio value is, the more organized the H-bond network. This innovative methodology, which undoubtedly enables the characterization of dipole-dipole interactions, hydrophobic micro-domains, and H-bonds in an aqueous medium composed of a saline phosphate buffer solution was then applied to investigate such molecular interactions in aqueous X/GM mixtures. The results exposed above demonstrate the existence of hydrophobic micro-domains, dipole-dipole interactions, and hydrogen bonds in X/LBG systems. These three types of interaction can occur either between both polymer chains (intermolecular interactions) or within each polysaccharide itself (intramolecular interactions), as is suggested from the results in the case of pure polysaccharide solutions. Molecular synergies for hydrophobic micro-domains and H-bonds were revealed only for a unique X/LBG ratio at 50/50. The EI_ANS_ and EI_1N*_/EI_T*_ ratios at this ratio show the highest values, which means that at this specific mixture composition, hydrophobic micro-domains and the H-bond network are, respectively, the most intense and the densest. However, despite their presence, dipole-dipole interactions clearly appear as background molecular interactions in X/LBG associations. Thus, dipole-dipole interactions may play a minor role in X/LBG mixtures’ properties if compared to both hydrophobic micro-domains and hydrogen bond interactions. This new methodology has proven its efficacy for an X/LBG system by applying strict conditions, such as a total polymer concentration of 0.2% (*w*/*w*). This innovative methodology was able to measure molecular interactions in a complex medium composed of polysaccharides and their solutions and can also be used as a new tool in order to better understand dipole-dipole interactions, hydrophobic micro-domains, and H-bonds. It can be used to identify these three types of molecular interactions, to measure the intensities of these molecular interactions according to the polymer mixture composition, to study the way they organize themselves, and to study their establishments through time in order to analyze how each type of molecular interaction can be affected by others (submission in progress). It can be applied to similar systems such as X/GG known to also exhibit a synergy of interaction at the macroscopic scale.

### 3.4. Influence of the Chemical Structures of Galactomannans

Both X and GM are well-known to exhibit a synergy of interaction in specific conditions. In the literature [50,57,58], it has been showed that stronger gels are obtained mainly when X in its disordered form (semi-flexible chains) interacts with GM, which has a few galactose residues, showing that the conformation and microstructure of these polysaccharides are important parameters. Based on macroscopic observation techniques, interaction models were proposed in the literature [57,59,60] to better understand X/GM associations in aqueous solution, but the authors did not come with the same agreement due to the structural variability of the xanthan and galactomannan samples used by the authors, leaving the mechanisms of interaction between both gums still misunderstood. However, attempts to elucidate these interactions were carried out at the molecular scale to know how and why X/GM mixture synergy occurs; for example, by studying the intrinsic viscosities of both gums and of their mixtures [61], by scrupulously characterizing X and GM gums [62], by studying aroma retention in X/GM matrices [63,64,65,66,67], and by performing 2D NOESY NMR experiments on X/LBG mixtures [68].

In the present work, in order to link together macroscopic (rheology) and molecular (fluorescence spectroscopy) observations, another type of galactomannan, guar gum (GG), was fully characterized from a structural point of view (GG: M/G = 1.4 and DB = 33%; Table 1) and mixed with the same xanthan gum (X) at a single mixture ratio (50/50). Locust bean gums (LBGs) are well-known to have a higher M/G ratio than guar gums (GGs) [69]. A comparison between both mixtures, X/LBG and X/GG, was made at a unique mixture ratio of 50/50 in order to determine the influence of the microstructure of the GM, although the X/GG mixture might not be at a maximum ratio. Another structural key property for galactomannans is the galactose residue distribution along the mannan main chain, defined as the degree of blockiness [70]. From a general point of view, LBGs with a high DB combined with a high M/G ratio generally have many more “smooth zones” (deprived of galactose unit) when compared to GGs, the latter having much lower M/G ratios. Storage (G′) and loss (G″) moduli values and the loss tangent (Tanδ) were measured once X/GM 50/50 mixtures were established at an equilibrium state (#48H) and are reported in Table 3. Tanδ is defined as the ratio of the G″ loss modulus over the G′ storage modulus (Tanδ = G″/G′) and indicate whether the studied sample adopts a predominantly elastic (G′ > G″) or viscous (G′ < G″) behavior. The X/LBG mixture undoubtedly develops much higher viscoelastic properties, as illustrated by rheological parameters in Table 3, with a storage modulus four times greater than the X/GG storage modulus. These results are in good agreement with the literature [57,58]. According to Table 3, one can observe that guar gum has strong structural differences, which clearly have an impact on the molecular interactions. Indeed, the EI of the ANS probe differs by 40 arbitrary units, meaning that hydrophobic micro-domains are much more present in the X/LBG mixture when compared to the X/GG mixture. Furthermore, the hydrogen bond network is also more developed; this result may be due to X, which preferably interacts with gum mannose residues that are less present in GG compared to LBG. Since LBGs and GGs have different microstructures, GGs are known to be more easily solubilized than LBGs, which means they are more hydrophilic polymers than LBGs. As mannose is an epimer of glucose with C2, mannopyranan polymer chains tend to form stable crystalline zones such as cellulose [71]. GGs possess more galactose residues along the mannosidic main backbone, thus limiting the occurrence of such crystalline zones by (1) limiting mannanpyran chain association and (2) providing more hydroxyl groups that increase the polysaccharide’s hydrophilic character. LBGs are, therefore, more hydrophobic than GGs, which may explain why hydrophobic micro-domains are more intense in the case of X/LBG systems than in X/GG systems. Concerning the hydrogen bond network, it can be hypothesized that X chains may interact preferentially with the smooth zones of GM mainly by developing hydrogen bonds between both gums. GGs, unlike LBGs, have less of these smooth zones, which could explain the different values of the EI_1N*_/EI_T*_ ratio between both systems studied. Finally, at the molecular level, the X/LBG system shows more intense interactions than the X/GG system, which reflects what we can observe at the macroscopic scale with the visco-elastic properties of such systems.

## 4. Conclusions

In this work, a new methodology based on the use of fluorescent molecular dyes, namely ANS and 3HQ-Bf, was developed to characterize at the molecular scale dipole-dipole interactions, hydrophobic micro-domains, and H-bonds for polysaccharide solutions and mixtures in aqueous media. In the first step, the behavior of both molecular probes was investigated, each taken separately, in a simple aqueous medium consisting of saline phosphate buffer to ensure accurate specific fluorescent responses according to their spectroscopic properties. All the parameters influencing the fluorescence properties were studied and optimized to ensure reliable results for the three specific interactions investigated. At the end of this first step and once the conditions of analysis were fixed, a new analytic methodology was proposed to make possible the characterization of dipole-dipole interactions, hydrophobic micro-domains, and the H-bond network at the molecular scale in an aqueous medium.

This methodology was then applied to xanthan (X) and galactomannan (GM) gum aqueous solutions and mixtures and proved to be efficient. Again, protocols were developed and optimized to overcome the limits; in particular, a strict protocol for the preparation of the polysaccharide solutions and mixtures, in the absence or in the presence of fluorescence probes, was developed, keeping the total polymer concentration lower than 0.2% (*w*/*w*).

This methodology, applied to aqueous X and GM gum solutions and mixtures, turned out to be very efficient. The three types of interactions were clearly identified for X/GM systems, but dipole-dipole interactions were demonstrated to not play a significant role in the foreground. Thanks to this innovative methodology, the roles of X and LBG at the molecular scale were brought to light; X clearly favors hydrophobic micro-domains, while LBG mainly acts as a hydrogen bond network promoter in X/LBG mixtures. A key result for the present study is that X/LBG mixtures do behave in a very specific manner at a 50/50 (*w*/*w*) mixture ratio; the present study clearly demonstrates that this specific X/LBG ratio exhibits both the most intense hydrophobic micro-domains and the most developed hydrogen bond network; this striking unpublished result demonstrates the existence of a simultaneous synergy at the molecular level for both interactions at this specific mixture ratio of 50/50. It is interesting to note that the macroscopic synergy for the same X/LBG system, as classically measured through rheological tools, indicated an optimum ratio of 40/60; therefore, the synergism of interactions may be interpreted in a different manner depending on the scale of observation.

To summarize, through an illustration for polysaccharidic solutions and mixtures, this paper describes a fully original tool to investigate molecular interactions in aqueous media. It is obvious that this methodology based on fluorescence measurements is reliable and efficient to characterize molecular interactions in polymer aqueous media. These first results are very promising and imply that this methodology may be extended to any polymeric systems (either natural or not) to characterize and understand the mechanisms involved for intra/intermolecular interactions at the molecular level.

## Figures and Tables

**Table 1:** Gum main characteristics.

Gum	M_v_^a^ ×10^6^	Protein (% *w*/*w*)	DS_Ac_ (%) ^b^	DS_Py_ (%) ^b^	T_m_ (°C) ^c^	M/G Ratio ^d^	Degree of Blocness (DB; %) ^e^
X	1.46	4.7	35	65	64 ± 1	/	/
LBG	1.6	1.22 ± 0.03	/	/	/	2.7	95
GG	2.67	1.33 ± 0.15	/	/	/	1.4	33

^a^ Determined by viscometric measurements with an Ubbelohde tube; ^b^ Determined by H NMR spectroscopy according to Esquenet’s protocol [38]; ^c^ Mean value calculated according to polarimetric and rheological measurements conducted according to Pelletier’s protocol [39]; ^d^ Determined by HPLC analysis according to Dahi and co-workers’ protocol [40]; ^e^ Calculated according to Tapie and co-workers [41].

**Table 2:** Examples of the raw EI values of the 1N* and T* spectral bands for X/LBG 60/40 at t = 24 h (extracted data of one of the three repeated experiences).

	1N* (λ_393 nm_)	2N* (λ_414 nm_)
Raw EI (a.u.)	179	190
	187	188
	182	177
	187	172
	186	191
Mean value	184 ± 4	184 ± 9

**Table 3:** Storage modulus (G′), loss modulus (G″), loss tangent (Tanδ), and spectroscopic data values of X/GM samples.

Samples	G′ (Pa)	G″ (Pa)	Tanδ	EI_ANS_ (a.u *)	EI_1N*_/EI_T*_
X	0.73 ± 0.10	0.59 ± 0.05	0.80 ± 0.03	365 ± 14	1.7 ± 0
LBG	2.4 × 10^−4^ ± 0.6 × 10^−4^	1.1 × 10^−2^ ± 0.1 × 10^−2^	49 ± 11	142 ± 13	2.3 ± 0.1
X/LBG 50/50	3.9 ± 0.7	0.60 ± 0.03	0.16 ± 0.02	323 ± 46	2.245 ± 0.382
X/GG 50/50	0.16 ± 0.01	0.27 ± 0.13	1.72 ± 0.94	280 ± 26	0.794 ± 0.064

* a.u. = arbitrary units of EI.

## Data Availability

The raw data supporting the conclusions of this article will be made available by the authors on request
